# Effects on nonverbal numerical acuity performance after a single-session transient random noise stimulation over the intraparietal sulcus or dorsolateral prefrontal cortex

**DOI:** 10.1038/s41598-025-21890-x

**Published:** 2025-10-30

**Authors:** E. Ó Dúinín, J. K. Steopan, K. Kessler, F. H. Santos

**Affiliations:** 1 School of Psychology, University College Dublin, Dublin, Ireland; 2 Institute of Education, University College London, London, UK

**Keywords:** tRNS, Mathematics, Dyscalculia, NIBS, Number line

## Abstract

We investigated the effects of cognitive training with transcranial random noise stimulation (tRNS) applied to the intraparietal sulcus (IPS) or dorsolateral prefrontal cortex (DLPFC) on improving a multidirectional number line estimation task. We predicted that a single session of tRNS targeting IPS would enhance accuracy without affecting response speed, while a single session of tRNS targeting the DLPFC would improve response speed without influencing accuracy. Through a repeated measure within-between-subject design, 39 healthy participants (M = 21.39, SD = 2.64) were divided into either an IPS (n = 20) or DLPFC (n = 19) bilateral stimulation group, whereby in a counterbalanced order, the participants received a sham session and a session of tRNS separated by 1 week. Stimulation was paired with training tasks focused on the approximate number system. Participants were measured by their speed and accuracy on a multidirectional number line estimation task. Findings partially support predictions, tRNS to the IPS improved accuracy but not speed on the number line estimation task after a single session. Contrary to expectations on tRNS to the DLPFC, no effects were observed. Findings contribute to our understanding of using a single session tRNS as a tool in interventions aimed at maths low achievers.

**Supplementary Information:**

The online version contains supplementary material available at 10.1038/s41598-025-21890-x.

## Introduction

Brains encode magnitude information by building upon phylogenetically pre-existing neural connections needed for species survival^1^. This is why numerosity is found in many animals, even those evolutionarily distant to humans, such as fish^2^ and birds^3^. These common neural circuits, which are considered inherent and typically observed in parietal structures, are said to underlie all numerical cognition, granting humans a ‘sense of magnitude’ that is required for mathematical literacy^4^. Although mathematics is associated with psychological wellbeing, career trajectory and socioeconomic status^5^, few neurocognitive interventions that augment numerical cognition exist. Such interventions would be highly beneficial for individuals with maths learning challenges, like developmental dyscalculia (DD), which is why transcranial random noise stimulation (tRNS) has recently become of increasing interest in the field of neuroscience as it is proposed to be the most effective form of electrical brain stimulation for improving accuracy and reducing reaction times in tasks utilising numerical cognition, such as arithmetic^5^.

The higher efficacy of tRNS could be due to the rapidly changing electrical field created by tRNS, which decreases the likelihood of membrane responses returning to a resting state^6^. tRNS is also less irritable and the physical sensations during stimulation are also harder to perceive compared to other electrical stimulation methods, such as transcranial direct current stimulation, making it an adequate experimental tool and promising candidate for intervention^5–7^. Although its exact mechanisms remain unknown, tRNS is a non-invasive technique that delivers a weak, stochastic current to the brain via scalp electrodes. This enhances cortical excitability in targeted regions, which, with appropriate cognitive training, can enhance neuroplasticity for an associated skill^8^. Studies applying tRNS have observed improvement in numerical abilities such as arithmetic^8,9^ and numerosity discrimination^7,10^. Transfer effects to untrained problems and long-term benefits have also been reported^5^, demonstrating tRNS utility for cognitive amelioration.

For instance, when applying tRNS paired with training over the parietal lobe of adults, Cappelletti and colleagues^7^ reported that the number acuity of stimulated participants improved significantly from baseline compared to control, with improved performance persisting up to 4-month follow-up. These results were further replicated in their later study, adopting the same paradigm^10^. There are also fMRI studies that observe the activation of the IPS during numerical tasks such as calculation, approximation and comparisons^9,11–13^. Additionally, fMRI studies indicate the recruitment of the DLPFC during arithmetic processes^12^, which is further corroborated by tRNS studies, which observe improvement in the speed of arithmetic skills post-DLPFC stimulation^8,9,14^, with Snowball et al.^15^ reporting enduring results (i.e., faster reaction times (RTs) on calculations) and transfer effects to untrained problems up to a 6-month follow-up for the stimulated group only. However, compared to the parietal lobe where the IPS is traditionally considered a hub for processing both symbolic and non-symbolic mathematics^11,16–18^, the role of the frontal lobe in numerical cognition remains uncertain and necessitates further exploration to clearly understand its role in the underlying numerical processes^5,12^. The present study targets these brain areas using a single session of tRNS in order to shed light on the efficacy of this paradigm for enhancing the underlying neural mechanisms of numerical cognition. Investigating the effects of only a single session of tRNS makes the present study unique in this field.

People process numbers in two ways, non-symbolically and symbolically. Non-symbolic numerical abilities refer to intuitive estimations of quantities known to operate using the Approximate Number System (ANS)^19^. Under Weber’s law, the ANS posits that quantities are ratio-dependant, becoming more distinguishable as the magnitude between them, such as size or distance, increases^20,21^. This skill is important for the development of our ‘number sense’, and studies observe that people who struggle to make visuospatial magnitude discriminations perform poorly in overall mathematics compared to peers as it is argued that the ANS and spatial skills share underlying constructs^18,22^. While non-symbolic abilities involve intuitive estimations of magnitude, symbolic abilities use symbols (e.g., the visual Arabic form) to complete more complex tasks, such as arithmetic^19,23^.

Some researchers consider that to gain meaning, symbolic numbers are mapped onto a linear representation of visual space, akin to a mental number line (MNL)^20,24^ which acts as a cognitive scaffold for several numerical strategies like estimation, counting and arithmetic^25^. The MNL consolidates the relationship between non-symbolic magnitudes and their symbolic counterparts and helps to refine our understanding of mathematics^24,25^. However, studies investigating whether training MNL estimations improves general mathematical abilities are inconclusive and research with adults is sparse because the MNL is often investigated concerning its role in the development of more general mathematical abilities in children^26,27^. Nonetheless, the MNL remains a subject of interest due to its contribution to our underlying sense of magnitude, which acts as a building block that eventually supports our ability to count and further perform more complex arithmetic problems with learning^20,24,25,27^. Therefore, the MNL should be especially considered when exploring the relationship between symbolic and non-symbolic abilities, the foundational skills of mathematics^25^. Understanding how these skills can be improved is particularly relevant as numerical processes are one of the most complex cognitive abilities humans possess, and arithmetic skills are crucial in day-to-day life^5^.

Therefore, observing the effects of tRNS on underlying numerical systems by increasing the cortical excitation of the IPS and DLPFC may not only shed light on the mechanisms involved in numerical cognition but may also have great practical implications for educational and clinical treatment interventions aimed at improving mathematical learning and psychological outcomes. This would be particularly relevant for individuals with DD who display deficits in these systems, as well as brain-related dysfunctions^22,23^.

Another factor reported to affect mathematical performance is maths anxiety. Maths anxiety can be described as persistent sensations of tension that impede the effective manipulation of numbers^22,28^. Additionally, maths anxiety is reported to hinder working memory capacity, which is arguably necessary for coordinating attention and problem-solving required in successful numerical cognition performance^14,22,28,29^. Some studies further report significant positive correlations between working memory and arithmetic^29^, making it a relevant factor to consider in tES interventions aimed at improving maths outcomes. Furthermore, gender is also worth accounting for as research suggests that females have a lesser response in their cortical excitability changes in response to tES, primarily due to skull and tissue differences, and hormonal changes and may also be more susceptible to developing maths anxiety^5,6,29^.

As such, the present study explored how symbolic and non-symbolic comparison task training paired with tRNS to the IPS or the DLPFC affects MNL processing, as measured by performance on a multidirectional number line estimation task^25^. A dissociation was expected whereby it was predicted that, (a) a single session of tRNS on the IPS would improve accuracy but not speed and (b) a single session of tRNS on the DLPFC would improve speed but not accuracy. Additionally, gender, baseline maths performance, maths anxiety and working memory were also assessed as exploratory covariates due to their potential effects on tRNS outcomes^14,28^. Therefore, investigating such effects in our study may provide direction for future interventions aimed at improving maths learning outcomes.

## Methods

### Experimental design

This study was a randomised controlled trial which adopted a 2 × 2 × 2 repeated measures crossover design. Participants were divided into two groups, those who would receive stimulation to the dorsolateral prefrontal cortex in the frontal lobe (DLPFC) (F3 and F4) or intraparietal sulcus in the parietal lobe (IPS) (P3 and P4), with group acting as the between-subjects factor. Both groups were assessed on a multidirectional number line task at two different time points (i.e., pre-, and post-treatment conditions). The groups underwent these treatment conditions in a double-blinded, counterbalanced order, either starting with the tRNS (treated) condition or with the sham (untreated) condition. Order of treatment condition functioned as the other within-subjects factor. Participants completed the magnitude comparison task during both sessions. A minimum of six days and a maximum of nine days (*M* = 7.45, *SD* = 1.1) separated the two sessions to avoid transfer of training^30^. Additionally, to control for confounding factors, participants were also assessed for working memory, math ability, and math anxiety prior to stimulation.

### Stimulation design

Participants were required to pass a pre-screening safety questionnaire in line with the University’s standard operating procedures (SOP) to determine eligibility. In the active stimulation session, tRNS was applied for 20 min, including a 30 s ramp up and 30 s ramp down. During the sham stimulation session, tRNS was only active for the ramp up and down period to elicit stimulation sensations and elicit a placebo effect. Participants were also required to fill out a comfort questionnaire by Antal et al.^31^ at the end of the sessions to rate their level of pain and report any abnormal sensations felt during the stimulation (see OSF files for these responses).

#### tES apparatus and application

The MXN-33/65 High Definition-Transcranial Electrical Current (HD-tES) Stimulator, Model 3200C, by Soterix Medical was utilized. A weak electrical current was transmitted on the scalp (F3 and F4 for the DLPFC group and P3 and P4 for the IPS group) using two HD electrodes with one acting as a reference channel, which were fitted on the participants’ scalp with conductive gel. This was done in accordance with the 10/20 EEG system. The protocol was double-blinded. Researchers, FS and KK, programmed the conditions on the HD-SC interface, which is the software associated with the tES apparatus, in such a way that the procedure could be run without the experimenters, JKS and EÓD, being aware of whether active or sham stimulation was occurring. As such, neither the experimenter nor the participant was aware of which treatment condition was being induced across sessions. After both sessions were completed, participants were debriefed and asked if they were aware of which session included the active tRNS stimulation. 49% of participants were correct, 43% were incorrect, and 8% were unsure, confirming that the sham was an effective placebo.

#### Stimulation parameters

The apparatus was switched to tRNS waveform and set to high frequency oscillatory direct current between 100 and 600 Hz for 20 min with a 30 s ramp up/down period at a 1μA intensity. These parameters are safe and in line with standard tES procedures^30^.

### Participants and recruitment

The present study recruited a total of 79 participants via poster advertisements, social media, and word of mouth. All participants were required to pass a pre-screening questionnaire to ensure their safety and determine eligibility. Participants with a history of traumatic brain injury or a neurological/psychiatric condition incompatible with stimulation (e.g., concussion, epilepsy, and depression) were ineligible to participate in the study. A total of 44 students were eligible for participation and took part in the experiment. However, as per the preregistration, participants with accuracy scores or reaction times more than 2.5 standard deviations from the mean were removed. This left the final sample, which consisted of a total of 39 eligible, healthy university students aged between 18 and 30 years old (*M* = 21.39, *SD* = 2.64, Male *n* = 19, Female *n* = 20).

Eligible participants were assigned to one of two stimulation groups: either the DLPFC group (*n* = 19) or the IPS group (*n* = 20) and these participants were pseudo-randomly assigned to either receive stimulation (*n* = 17) or sham (*n* = 22) in their first session. Age and gender were relatively equally distributed across groups (See Table 1). Informed consent was gathered online prior to data collection and participants were monetarily compensated for taking part (i.e., a OneForAll voucher).

**Table 1:** Age and gender descriptives for subgroups.

		Age		Gender
		*M*	*SD*	(Male %)
Brain area	DLPFC	21.52	2.96	47
	IPS	21.25	2.35	50
Order of treatment	Sham 1st	22.22	3.13	50
	tRNS 1st	20.29	1.21	47

### Experimental stimuli

#### Spatial numerical acuity

To assess the reaction time and accuracy of spatial numerical associations, a multidirectional number line (MNL) task - adapted from Leonard et al.^25 ^and previously tested in university students^32^, was modified for the experiment under the authors’ oversight. This task was applied pre and post treatment condition during both sessions using Gorilla Experiment Builder (https://gorilla.sc/). The MNL contained 48 randomised trials presenting participants with Arabic numbers ranging from 0 to 100 and instructing them to make number line estimations on a laptop using a mouse. The number line trials varied across four modalities left–right or right-left when horizontal, and top–bottom or bottom-top when vertical. There was a break displayed on screen midway, and stimuli were presented in black against a white background, with the number line consistently measuring 1200 pixels in length across trials.

**Fig. 1:**
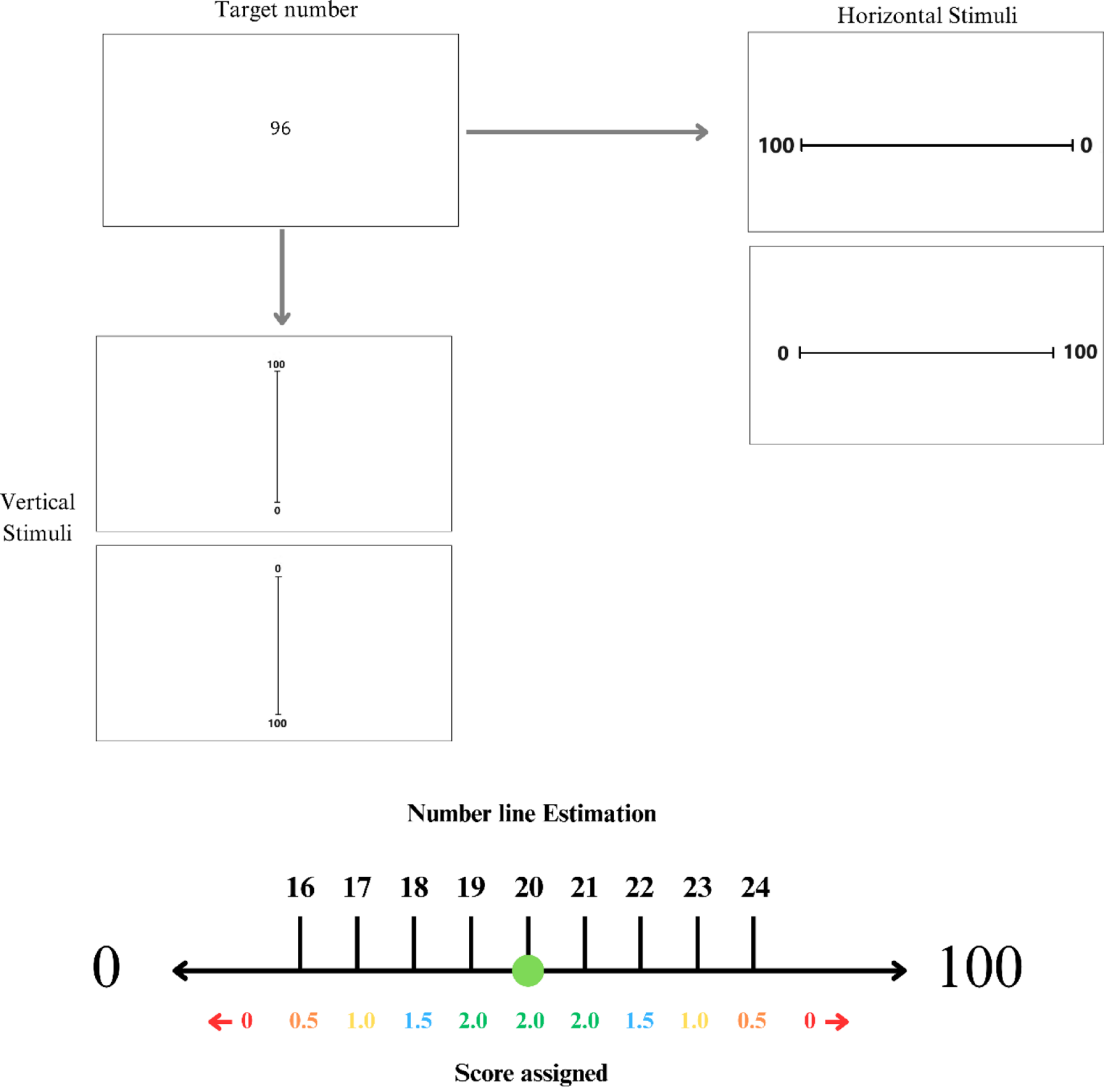
Vertical and horizontal stimuli for number line task and scoring process for the MNL where the target is 20.

To score accuracy for the MNL, Kucian and colleagues’^23^ point system was used. This was done by assigning a score to the precise x and y screen coordinates participants placed the mouse on to indicate a response. This point system ranged from 0 to 2 whereby a response placed two whole numbers before or after the target answer scored 2, a response between two to four whole numbers scored 1.5, and a response that measures eight whole numbers away from the target answer scored 0 points. Refer to Fig. 1 for a visual of the point system. Additionally, reaction times (RTs) for this task were measured in milliseconds (ms) and a unique code was assigned to participants in Gorilla (e.g. ID012) to link corresponding data.

### Magnitude discrimination training

Participants were presented with two training tasks via PsychoPy (https://www.psychopy.org/), (a) a symbolic magnitude comparison task and (b) a non-symbolic dots discrimination task. Each task was programmed on PsychoPy and presented on a HP laptop, lasting approximately five minutes each to cover the last 10 min of stimulation during both tRNS sessions, beginning with the symbolic magnitude comparison task, and followed by the non-symbolic magnitude comparison task. Keeping to the same ratio of trial congruencies, both tasks were shortened in order to fit into the current paradigm. The training tasks were preceded by a practice session prior to stimulation, which included three trials of the symbolic and non-symbolic tasks, which participants could repeat until they understood the task.

*a) Symbolic–Magnitude Comparison Task:* An adapted version of Sasanguie et al.’s^33^ (also see^34^) numerical comparison task was used whereby only the symbolic set of stimuli was utilised. Participants were presented with instructions followed by a fixation cross for 600 ms and were then required to make numerical discrimination judgements between two Arabic numbers using the left and right keys on the keyboard. The numbers ranged between 1 and 9, with a maximum difference of five, and the task contained a total of 72 randomised trials, which were presented in black text on a light grey screen. Accuracy and RTs were measured for this task.

*b) Non-Symbolic–Dots Discrimination Task:* An updated dots discrimination task was provided to the experimenters by the author (see^35^), which presented participants with two arrays of dots, one set in blue and another set in yellow. Participants were required to identify which array had more dots by clicking either ‘b’ (for blue) or ‘y’ (for yellow) on the keyboard. There was a total of 130 randomised trials in this task, and the quantity of dots in the arrays ranged across a ratio of 1.12, 1.2, 1.3, 1.5 or 2. This meant that one array always had 16 dots while the other had between 8, 11, 12, 13, 14, 18, 19, 21, 24 or 32 dots. All trials were also equally spread across the four types of congruences as defined by DeWind et al.^36^: fully congruent, fully incongruent, spacing congruent but size incongruent, spacing incongruent, but size congruent. Total accuracy and RTs across congruency types were measured for this task.

### Exploratory variables

### Maths Anxiety

Participants completed the short 9-item Abbreviated Math Anxiety Scale (AMAS) designed by Hopko et al.^37^. This was completed online via Pavolvia (https://pavlovia.org/) and participants were required to indicate their level of maths anxiety by rating statements on a 5-point Likert scale ranging from ‘low anxiety’ to ‘high anxiety’. This measure comprises two subscales (Maths Learning Anxiety with five items and Maths Evaluation/Testing Anxiety with four items), which previous studies have reported strong internal consistency (α = 0.90) and good test–retest reliability (*r* = 0.85)^37,38^. Total scores are then calculated, where higher scores on this scale are indicative of higher levels of maths anxiety and vice versa.

#### General arithmetic skills

Participants were required to complete the 2-min-Speed Test in Arithmetic adopted from Artemenko and colleagues^39^ (see also^40^) study to assess baseline maths performance. Participants had a total of two minutes to complete a mix of as many addition, subtraction, multiplication, and division problems as they could, with a maximum of 40 calculations possible. Calculations were presented with Arabic numbers with simple and complex trials. Simple addition and subtraction trials did not require the carry over or borrow procedures common in arithmetic, whereas complex trials did. For the subtraction trials, the inverse of the addition calculations was used (e.g., 25 + 51 = 76 → 76 − 51 = 25). Simple multiplication trials included a problem with two single-digit operands (between 2 and 9), while complex trials had a single-digit operand paired with a two-digit (between 12 and 19) operand. For the division trials, the inverse of the multiplication calculations was used (e.g., 9 × 7 = 63 → 63 ÷ 7 = 9). The stimuli were presented in a randomised order, one at a time, as such participants could not see the next calculation until they solved the current one on screen. A higher score of correct responses is indicative of better maths performance and vice versa.

#### Working memory

A N-back (2-back) test designed in PsyToolKit Version 3.4.6^41,42^ was used to measure participants’ working memory skills. Participants were presented with a stream of disappearing letters, which they were required to hold in their short-term memory in order to identify if the target letter matched the previous letter they saw two stimuli ago. Participants were presented with three blocks of 25 trials each, whereby the first block was a practice trial. Each stimulus remained on screen for 760 ms at 2000 ms intertrial intervals with a total of 15 stimuli randomly presented across the blocks using a combination of the letters A,B,C,D,E,H,I,K,L,M,O,P,R,S, and T. Participants were required to click the letter onscreen with a mouse to indicate a correct response. Accuracy and RTs were measured.

### Procedure

Prior to the study, participants were pre-screened on Google forms. An email link was sent to eligible participants to obtain informed consent through Pavlovia, which then redirected them to the maths anxiety questionnaire and maths performance task to be completed personally, online under their unique ID code. Upon arriving at the lab, the tRNS equipment was installed on their scalp by the experimenters while they filled out the second part of the screening, inquiring about their recent water/food intake as well as consumption of caffeine, drugs or alcohol. Participants were asked not to drink coffee or other stimulants prior to participation and the consumption of which would have required them to reschedule. This action was never required. Participants were informed in the consent form and again in person that they would undergo two sessions of tRNS, one week apart.

The two sessions were double-blinded and operated in a counterbalanced order where half of the participants began with the active tRNS condition and the other half with the sham condition, equally dividing participants in IPS and DLPFC groups. In the first session, participants were seated comfortably at a desk with ceiling lights on, facing a wall without a view of the experimenters or a window in order to minimise distractions. They were encouraged to relax and remain still while attached to the equipment. All tasks were completed on a 14″ laptop. Participants were first required to complete the working memory task, followed by the MNL, which offered them optional breaks. After this, participants had the opportunity to practice the training task, and once this was finished, they were instructed to close their eyes and told that stimulation was set to begin. For the first five minutes of stimulation, participants were kindly instructed to close their eyes and sit still in silence, whereas at the five-minute mark, they were asked to open their eyes. Participants were given periodic updates as time passed for the first 10 min. Once this time elapsed, participants were given the training task for the last 10 min of stimulation, adding up to a total of 20 min. Once the stimulation block was over, participants were instructed to complete the MNL again, which concluded the experimental component of session one. Before leaving the lab, participants completed a comfort questionnaire to indicate their sensations under stimulation, offered a free coffee and scheduled for their second tRNS session. The first session lasted between 40 to 45 min.

For session two, the same protocol as session one was repeated, excluding the exploratory measures tasks, and adding a debrief paired with a questionnaire asking participants when they thought they received sham vs stimulation. The second session lasted between 30 to 35 min. At the end of the study, participants were debriefed, given supports, and monetarily compensated for taking part. The total study was approximately 70–80 min.

### tRNS safety measures

While tRNS is safe and non-invasive, participants may still have negative physiological experiences during active stimulation. Research has shown no signs of any major issues arising from the stimulation in human trials. To ensure the safety of all the participants, the protocol remained within the parameters set by previous tES studies^31^, keeping the current to less than 4 milliamperes and the stimulation time under 40 min. Some rare cases of adverse physical sensations, such as burning and irritation to the scalp, have been recorded in previous research. To avoid this, it was ensured that the areas of contact with electrodes were suitable (no cuts or rashes etc.) and sufficiently prepared, filling with the saline gel solution before stimulation began. Participants completed the ‘Questionnaire of sensations related to transcranial electrical stimulation’^31^ to allow them to share any discomfort experienced. Participants were given a five-minute break after stimulation, and they were provided with information on how to reduce potential side effects. Following the University’s SOP, participants were encouraged that if side effects continue for longer than 24 h, they should visit their general practitioner. There were also check-ins at regular intervals with the participant during the stimulation, asking if they were happy to continue, as recommended in the University’s SOP for tES.

### Data analysis

Data were pre-registered and analysed using SPSS 27. To assess whether parametric tests could be used, the data were first inspected for approximate normality by observing Q-Q plots. Shapiro-Wilks test of normality was then conducted, and skewness and kurtosis were observed. Paired sample t-tests were performed to analyse whether means were different before and after the stimulation (or sham) in each session. The accuracy scores and reaction times pre-stimulation (or sham) for the first and second sessions were also compared using a t-test to establish whether the baseline performance was significantly different in either session.

To test the main hypotheses, a two-way ANCOVA was conducted, with accuracy or reaction time (taken from the MNL) as the dependent variable, and brain area, time, and treatment as the independent variables. There were two levels to each of the independent variables: time was labelled as pre and post; brain area as frontal and parietal lobe; and treatment was labelled as sham and stimulation. The covariates included in the analysis were gender, working memory, maths ability, and maths anxiety. Significant interactions found between time, treatment and brain area stimulated in the ANCOVA were further analysed using pairwise comparisons. Deviating from the preregistration, the analysis was repeated with the covariates removed, to ensure the data was not being overfitted.

The continuous variables (working memory score, working memory reaction time, maths ability, and maths anxiety) were also tested for normality using the Shapiro–Wilk test and by examining the skewness and kurtosis of the data. To ensure internal validity within the data collected, various tests were conducted on relevant variables to test for expected relationships and increase the likelihood of the analyses being representative of an accurate population effect. The relationship between maths anxiety and maths ability was tested using a Pearson’s correlation. The differences in maths anxiety and maths ability between genders were also tested using linear regressions. The relationship between working memory and maths ability was also tested using a Pearson’s correlation.

To assess the effects of these variables on tRNS, the interactions between covariates and treatment from the same 2 × 2 × 2 ANCOVA were observed. Deviating from the preregistration, any variables with significant interactions with treatment were further analysed by separating them into categorical variables and running pairwise comparisons.

#### Adherence to relevant guidelines/regulations

All methods were carried out in accordance with the declaration of Helsinki Ethical Principles for Medical Research involving human subjects and the university’s SOP for tES. Tissue samples were not collected as the study involves tRNS which is a non-invasive brain stimulation technique.

#### Informed consent

Prior to participation in this study, informed consent was collected from all participants electronically.

## Results

### Data management

The data used in this study were collected from six measures. For the multi-directional number line task, scores were assigned to each trial according to the description in the methods section and then summed to give a total accuracy score. Reaction times were also recorded for each trial and average reaction times were calculated for each participant. For the 2-Back task, incorrect responses were deducted from the total number of trials to give a total score. To represent the reaction times for the working memory task as accurately as possible, reaction times were only taken from the trials in which participants actively made a response, correct or incorrect (rather than inhibiting response), and then the average was calculated from these data points. For the symbolic and non-symbolic magnitude comparison task, total correct key responses were counted to give the total score, and average reaction time was calculated. Total correct responses were summed from the maths ability task to give a mathematical ability score. Total score from the AMAS was calculated to give total levels of maths anxiety. For further analysis, maths anxiety was also categorised into groups. Low and high were scores one standard deviation below and above the mean, respectively, as seen in previous studies^29^. Meaning for this study, participants with scores between 9 and 13 on the AMAS were classified as low MA (n = 9), moderate scores were 14–26 (n = 22), and high scores were of 27 and above (n = 8). Participants were excluded according to the criteria set in a preregistration, which are described in detail in the Methods section.

### Comparing means of accuracy and reaction time from the number line task

**Fig. 2:**
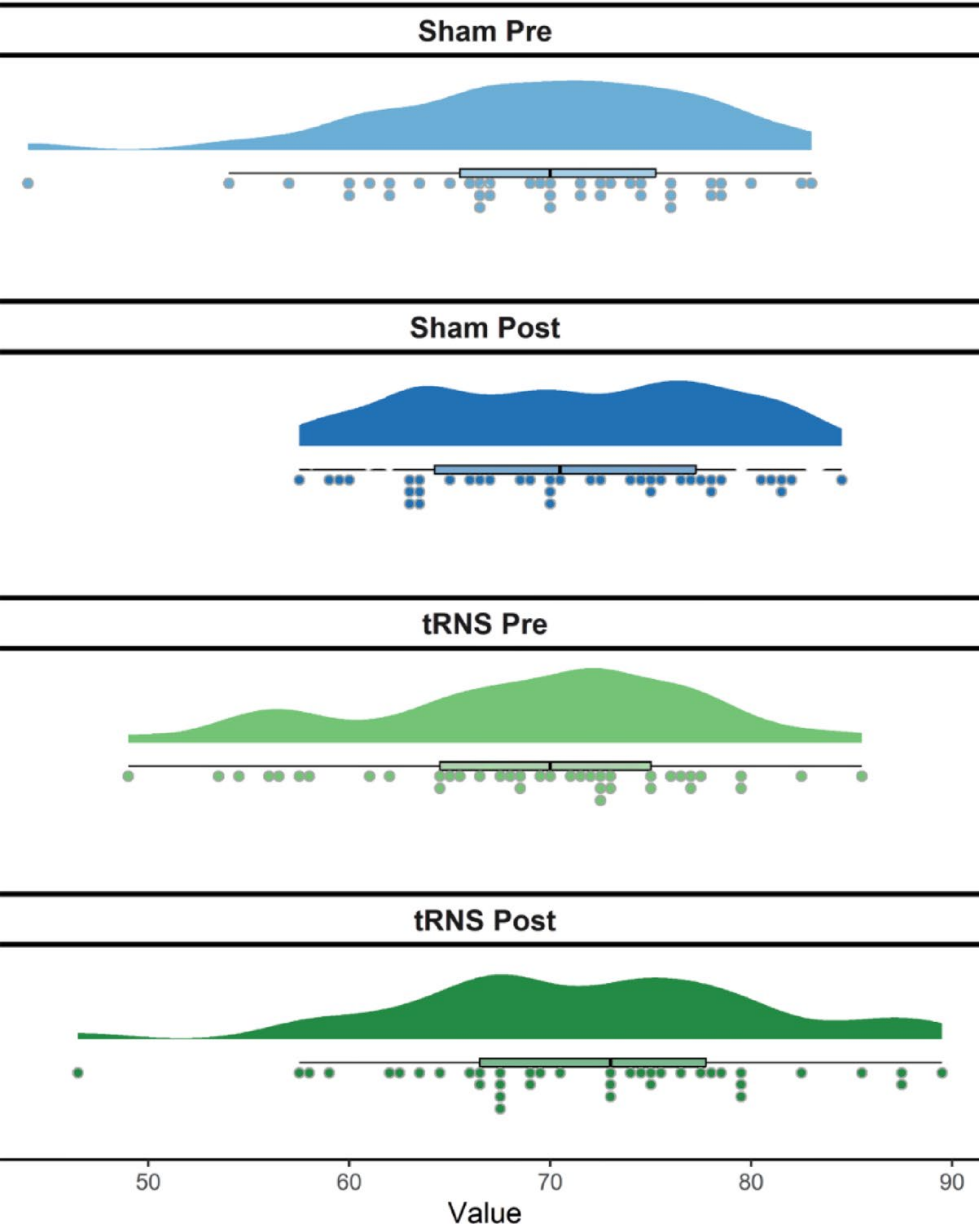
Raincloud plot of accuracy across treatments.

To first assess whether there was a learning effect, or whether there was a baseline difference between the treatment conditions (tRNS vs sham), paired sample t-tests were performed using the total accuracy scores and reaction times from the number line task, as calculated in the data management section (See Fig. 2 for the raincloud display of accuracy). The paired samples t-tests revealed that accuracy scores increased from pre to post in both the sham and the stimulation session (*t*(38) = − 1.8, *p* = 0.04, t(38) = − 2.546, *p* = 0.008, respectively) but that the baseline accuracy was not significantly different between the first and second session, *t*(38) = 0.298, *p* = 0.77. The paired sample tests revealed the same pattern for reaction times, where pre to post in each session improved (sham, *t*(38) = 3.32, *p* = 0.01; tRNS, *t*(38) = 3.45, *p* = 0.01), but baselines were not significantly different from each other, *t*(38) = 1.693, *p* = 0.09. These results show that while the baseline accuracy scores and reaction times did not change significantly from the sham to the stimulation session, participants’ performance was improving from pre to post during each session, although the specific effects of tRNS are yet to be explored.

### Investigating effects of treatment condition and brain area stimulated on number line performance

As preregistered, to assess the influence of the covariates on the effectiveness of tRNS, a repeated measures ANCOVA was conducted with working memory, maths ability, maths anxiety, and gender as covariates. See Table 2 for descriptives and mean differences of brain areas in each session. Residual normality was confirmed by observing Q–Q plots of the standardised residuals for each of the accuracy and reaction time datasets from the number line task. Shapiro-Wilks tests further confirmed the normality of the distribution of residuals with all *p* > 0.05 (See Supplementary materials). By testing the means and by observing grouped scatterplots of dependent and independent variables, covariates were all found to be linearly related to the accuracy and reaction times from the number line task. Covariates were not strongly correlated, with only maths anxiety and maths ability weakly negatively correlated (*r* = − 0.340, *p* = 0.015). Homogeneity of slopes was confirmed (*p* > 0.05). A Levene’s test revealed that ‘tRNS Pre’ did not display equality of variance (*p* = 0.042), but that all other dependent variables had equal variance between the brain areas stimulated. Despite this, ANCOVA is still an appropriate test for this analysis, though results may be slightly less reliable. To control for the increased risk of Type I error due to multiple comparisons, the Bonferroni correction was applied.

**Table 2:** Descriptives and paired sample differences in accuracy and reaction times.

		Accuracy		Reaction time	
		Mean	SD	Mean	SD
Sham Pre	Parietal	67.63	2.01	3203.35	183.98
	Frontal	71.29	1.58	3549.23	208.81
Sham Post	Parietal	69.55	1.87	2795.97	165.52
	Frontal	72.74	1.39	3403.3	227.74
tRNS Pre	Parietal	67.02	2.05	3178.17	171.58
	Frontal	70.76	1.74	3977.27	330.41
tRNS Post	Parietal	70.45	2.29	2835.11	126.49
	Frontal	71.89	1.54	3514.27	235.92
S1 Pre	Parietal	68	2.16	3238.54	198.74
	Frontal	70.82	1.66	3942.01	280.26
S2 Pre	Parietal	66.65	1.88	3142.98	153.52
	Frontal	71.24	1.67	3584.49	275.26
Sham Pre–Sham Post	Parietal	-1.93	6.21	407.37	441.65
	Frontal	-1.45	5.65	145.94	584.73
tRNS Pre–tRNS Post	Parietal	-3.43	5.53	343.05	605.79
	Frontal	-1.14	5.69	462.99	849.3
S1 Pre–S2 Pre	Parietal	1.35	5.45	95.56	905.86
	Frontal	-0.42	6.89	357.52	1156.71

The groups are labelled as follows: ‘Sham Pre’ = Task completed before the sham session; ‘Sham Post’ = task completed after the sham session; ‘tRNS pre’ = task completed before the stimulation session; ‘tRNS Post’ = task completed after the stimulation session. The means of the results before the first (‘S1 Pre’) and second session (‘S2 Pre’) were also compared to assess whether there was a different baseline between the sessions.

**Fig. 3:**
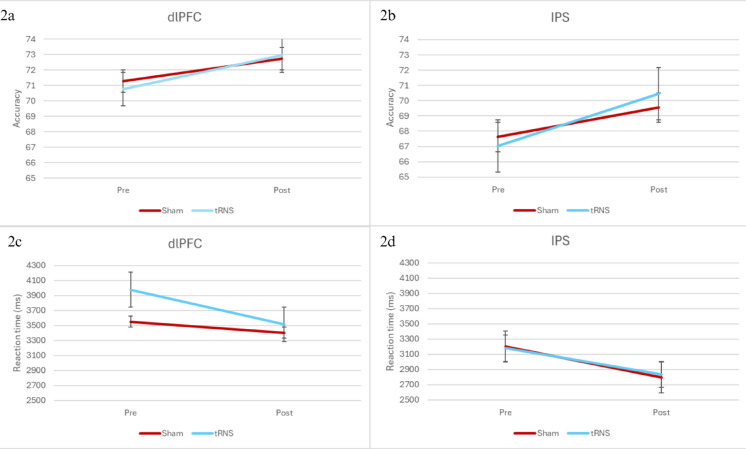
Interaction plots of Estimated marginal mean scores and error bars of accuracy and reaction time on MNL between time and treatment, separated by brain area stimulated. Error bars represent standard deviation.

No significant interaction was observed for accuracy between time, treatment, and stimulated brain area, while accounting for covariates, *F*(1,31) = 0.508, *p* = 0.481. See Fig. 3 for the display of this interaction. Despite the significant time differences observed from the t-tests conducted on the data from pre and post stimulation attempts, this analysis discovered no such main effect, *F*(1,31) = 1.734, *p* = 0.198. This would indicate that the difference observed using the t-test was at least partially explained by at least one of the covariates. There was also no main effect for treatment *F*(1,31) = 0.103, *p* = 0.750. Together, these results indicate that our hypothesis, that stimulation to the bilateral IPS would improve accuracy on the number line task, cannot be accepted for this study.

No significant reaction time interaction was found for time, treatment, and stimulated brain area, controlling for covariates, *F*(1,31) = 0.441, *p* = 0.512. See Fig. 3 for the display of this interaction. Despite the significant differences found by the t-test between the pre and post stimulation, the ANCOVA found no such main effect of time, *F*(1,31) = 0.652, *p* = 0.425, which means the variance found in the t-tests may be explained by one or more of the covariates. There was, however, a significant main effect of treatment, *F*(1,31) = 4.401, *p* = 0.044, which would indicate a difference in speed between the two sessions. However, this result is unclear as there were no significant differences in the treatments for time 1 and 2, when investigated using a pairwise comparison (*p* = 0.086; *p* = 0.342). Even with a significant treatment main effect, these results give us reason to dismiss our hypothesis that tRNS to the DLPFC would decrease reaction times.

Post-hoc power analyses revealed that this study only reached a power of 0.23 for accuracy and 0.14 for reaction time, so to confirm that these results were not caused by overfitting the data with only a small sample size, the covariates were removed, and the tests were repeated. However, still no significant interaction between time, treatment and brain area stimulated was observed for either accuracy *F*(1,37) = 0.503, *p* = 0.483, or reaction time, *F*(1,37) = 1.548, *p* = 0.221. These tests did show a main effect for time, with improvement for both accuracy (*F*(1,37) = 8.767, *p* = 0.005) and reaction time (*F*(1,37) = 25.861, *p* < 0.001) between attempts within sessions, confirming the t-test findings.

### Follow-up analysis: simple comparisons

**Fig. 4:**
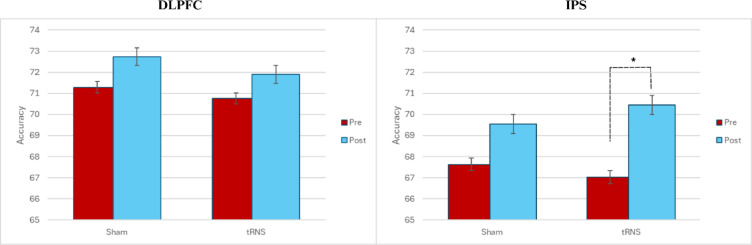
Mean accuracy differences between pre and post stimulation or sham. *The mean difference is significant at the 0.05 level. Error bars represent standard error.

**Fig. 5:**
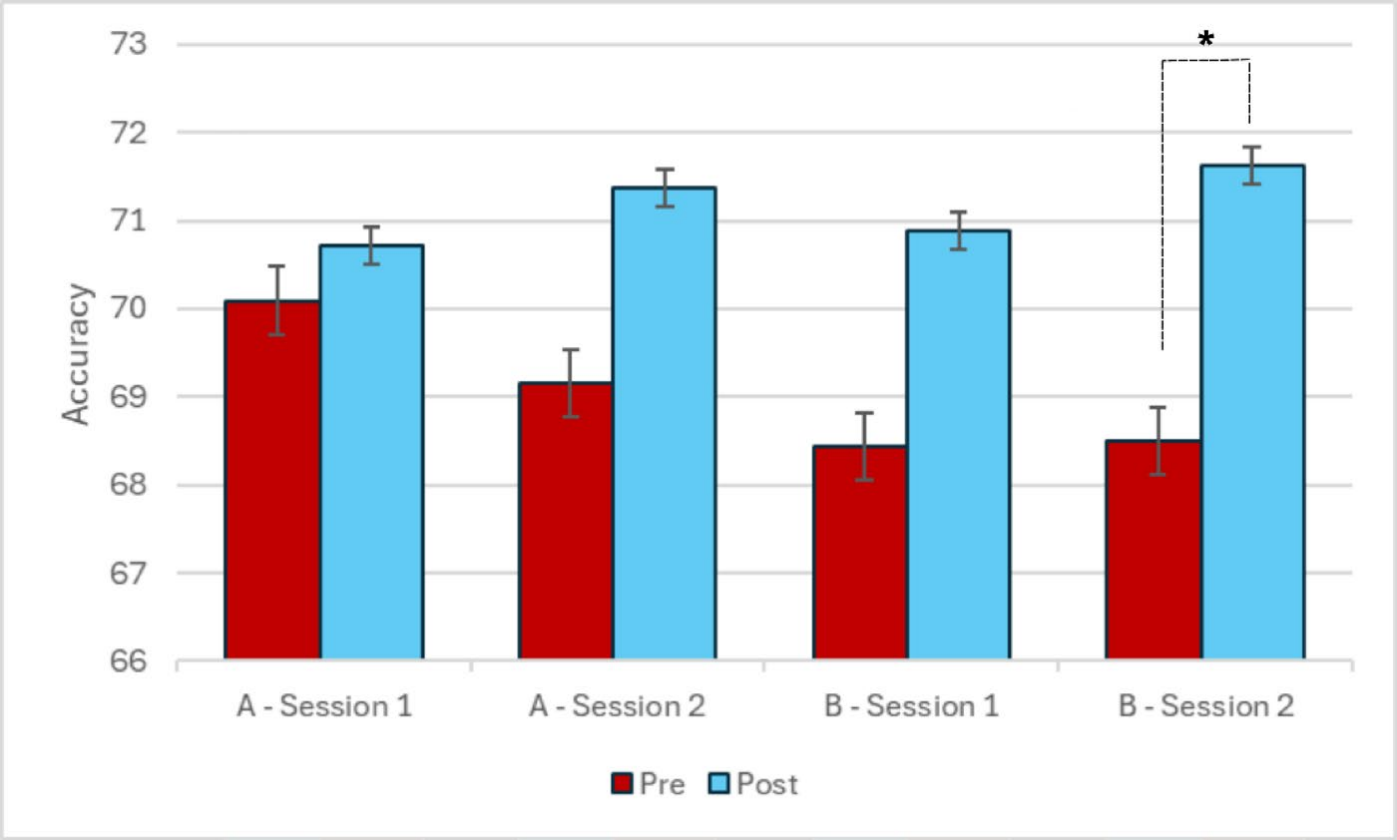
Mean accuracy differences between treatment conditions in sessions 1 and 2. *The mean difference is significant at the 0.05 level. Group A = Received sham tRNS in the first session. Group B = Received tRNS in the first session. Error bars represent standard error.

Deviating from preregistration, when exploring the effects of tRNS stimulation separately for each stimulation site, we observed that tRNS to IPS in fact led to a significant improvement in accuracy between pre and post (*p* = 0.012), as predicted (See Fig. 4). No such improvements were found for tRNS to DLPFC or for the sham conditions. Further analysis revealed that when the order of treatment condition was explored as a factor, the second session had improved accuracy between pre and post for the group who received tRNS to either the IPS or the DLPFC in the first session, despite the stimulation in the second session being a sham (*p* = 0.038) (See Fig. 5). No other comparisons revealed a significant difference.

Post-hoc testing for reaction times using pairwise comparisons revealed that when taking brain areas stimulated and order of treatment conditions as lone factors, as was done for accuracy, there were many significant improvements over time, but none of these improvements happened in a way that would indicate an effect of tRNS, or the brain area stimulated. Pairwise testing of the order of treatments as a factor revealed that when stimulated in the first session, this led to improved reaction times between pre- and post in both the first and second sessions.

### Follow-up analysis: covariate interactions with main effects

Using t-tests, it was established that there was a difference between pre and post tRNS, for both accuracy and reaction time on the number line task, however, the ANCOVA revealed that this effect may be attributed to other variables. There was a significant interaction between time and maths anxiety for accuracy (*F*(1,31) = 4.845, *p* = 0.035), indicating that maths anxiety is an important predictor for improvement in accuracy. Further analysis was conducted on this relationship, as preregistered, whereby the participants were separated into low, medium and high maths anxiety groups as described in the data management section, and conducting a pairwise comparison for pre vs post, it was seen that while low and medium maths anxiety scorers improved significantly between the two sessions, the high math anxiety group had no significant change (See Table 3 for details). This indicates that as maths anxiety increases, it adversely affects performance improvement on the number line task. There were no other relevant covariate interactions.

**Table 3:** MNL accuracy Pairwise Comparison per maths anxiety groups and time interaction.

Maths anxiety	Low	Medium	High
Time mean difference (1–2)	− 3.194*	− 2.284*	0.125
Sig	0.026	0.014	0.932

*The mean difference is significant at the 0.05 level.

## Discussion

The present study investigated the paired impact of tRNS in the IPS or DLPFC with symbolic and non-symbolic magnitude training on underlying processes involved in MNL estimations. It was hypothesised that 1) tRNS to the bilateral IPS would improve accuracy on the MNL but not speed, and that 2) tRNS to the DLPFC would decrease reaction times on the MNL with no direct effect on accuracy.

### Parietal tRNS enhances accuracy of underlying numerical systems, but frontal tRNS has no effect on speed

Our results indicated that a single session of tRNS did not significantly improve accuracy on the MNL regardless of the brain region stimulated, when controlling for the covariates of maths anxiety, gender, working memory and maths performance. However, the findings were expanded by the results of a pairwise comparison, which revealed that, when grouped by brain area stimulated, active tRNS to the parietal lobe significantly improved accuracy from pre to post stimulation, whereas no other groups showed significant improvement (See Fig. 4). This partially supports our hypothesis. Our findings contribute to existing literature with previous studies also identifying tRNS on the IPS as an effective method of improving numerical cognition^5,9,10,43^. Additionally, compared to other stimulation studies which targeted specific mathematical skills like arithmetic^4^, our study used the MNL task to explore magnitude discrimination in order to understand underlying mathematical abilities in adults, which are an under-researched cohort in this domain^25^.

Additionally, when participants were grouped by order of treatment (i.e., whether they received tRNS or sham in the first session), the group that received stimulation in the first session showed improved learning in the second session, despite this session being a sham (See Fig. 5). This indicates retention of a learning effect caused by tRNS. This replicates findings of previous studies, which have shown that even when performance does not improve immediately after stimulation, offline practice sessions can lead to improved performance^14,44^. This has further positive implications for the use of single sessions of tRNS as an intervention to improve numerical acuity.

Furthermore, results indicated that reaction times were not affected by tRNS regardless of the brain area stimulated and even after grouping by brain area or order of treatment. This dismisses our second hypothesis. This outcome was unexpected as past literature, such as that of Bieck and colleagues^8^ that targeted mental addition or Pasqualotto^14^ who investigated arithmetic skills, suggests that stimulation to the DLPFC improves speed, with several other studies reporting immediate and long-term effects here compared to sham^5,15^. Nonetheless, because reaction times were unchanged, this also allows us to dissociate between the functions affected by IPS stimulation (e.g., the IPS is directly involved in processing spatial numerical precision, but likely not in speed) and may be indicative of a speed-accuracy trade-off^5^.

Lastly, tRNS efficacy was not affected by either maths anxiety, maths ability or working memory and gender differences were not observed in our study, dismissing our exploratory hypotheses. However, a negative correlation between maths anxiety and maths ability was identified^28,29,45,46^. This is in line with past studies, as highly maths anxious individuals show lower numerical acuity^25,29^. Furthermore, participants with higher maths anxiety displayed no improvement in the MNL. Research has suggested tRNS can be more effective in those with maths anxiety due to changes in cortisol levels and that the IPS is a particularly appropriate target for the treatment of maths anxiety due to studies reporting cortical differences in the left IPS in those with maths anxiety^45,46^. These previous findings being considered, the lack of effect from maths anxiety is then likely due to the current sample being underpowered. Therefore, this factor, among others, should still be considered when developing treatment interventions aimed at improving mathematical performance in those with developmental dyscalculia or other intellectual difficulties.

### Exploring the lack of DLPFC stimulation effects and explaining limitations

#### Stimulation site

The lack of effect from tRNS to the DLPFC, could suggest that the MNL task we adopted relies more heavily on skills like processing and spatial awareness to perform MNL estimations and is therefore designed to target parietal structures (i.e. the P3 and P4) leading to a decrease in frontal activation, or at least specifically in the recruitment of the F3 and F4 regions that we targeted in our study^47,48^. For instance, neuroimaging studies observe that prefrontal areas are initiated earlier in mathematics when domain general resources like attention and working memory are recruited^5,23,48^. Therefore, the lack of stimulation effect observed in the DLPFC may be because our participants’ numerical acuity is matured and therefore sophisticated in parietal structures. Alternatively, although studies have demonstrated that electrical brain stimulation to the DLPFC improves speed and automaticity, it can also produce paradoxical effects, including impairment in specific cognitive processes needed to perform tasks^49^. It is possible that this paradoxical effect impaired the improvement to automaticity in the present study.

The increased variability from using the 10–20 EEG guide, should also be considered as a limitation of the present study. The system’s high variability across individuals can lead to imprecise stimulation of brain regions during tRNS, reducing the effectiveness and consistency of stimulation^50^. It should also be noted that future studies should aim to further account for this individual variability by modelling using individual MRIs^50^. This cannot, however, entirely explain the results found in the present study. Studies using other kinds of electrical brain stimulation, such as transcranial direct current stimulation, have used similar montages and found positive results^31,49,51,52^.

#### Training task difficulty

Some researchers argue that tRNS is more effective in training paradigms aimed at enhancing learning, rather than modulating cognition itself^8^. As such, the present findings may be explained by the *nature* of the training that participants received during stimulation (e.g., dots discrimination) rather than the main MNL task. Researchers propose that there is a minimum ‘*desired difficulty*’ level required to optimise the transfer of knowledge from cognitive training to the tested task, which increases excitation to relevant brain regions^9,15^. Researchers report that stimulation is most effective at improving accuracy and RTs during an extreme when the task is most difficult (i.e., more numerous, less repetitive), with tRNS mitigating task difficulty compared to sham and also being transferable to new, untrained arithmetic problems^9,10,15^. Even though dot discrimination has been used in university students^13^, our chosen stimuli may not have been challenging enough to activate our targeted brain regions, or a single session of tRNS paired with training may be insufficient to observe our expected results^5,8,9^. This pattern has been identified in previous studies, where tRNS to the DLPFC improved accuracy for children with dyscalculia, but only after multiple sessions^53^.

### The clinical significance of our findings and direction for future research

The positive effect on MNL accuracy when tRNS is accompanied by symbolic and non-symbolic magnitude comparison training gives further evidence of the relation between the two kinds of tasks and further highlights the potential benefits of tRNS in increasing transfer effects across cognitive functions. This finding suggests that ANS processing is centralised in the IPS and underlies magnitude representation and aspects of spatial numerical cognition. These findings open possibilities for interventions targeting those with specific numerical difficulties by allowing ANS-related tasks to be improved symbiotically.

The present study gave indications that a single session of tRNS paired with cognitive training to the IPS may be beneficial for improving tasks which rely on the ANS. Although the ANS is foundational to many aspects of numerical cognition, and improvements to its processes may indirectly benefit general mathematical abilities, researchers should explore its use in directly benefiting those more general abilities. This is particularly important for equalising the learning environment for those with higher levels of maths anxiety and developmental dyscalculia. A limited number of studies investigating tRNS and dyscalculia indicate that it is an effective method of strengthening learning effects in children with learning disorders^49,53^. The present study corroborates these studies in finding that a single session of tRNS can be effective in improving maths learning even in healthy adults.

It should be acknowledged that although the main interactions of interest were not significant, the presence of a simple effect of tRNS on accuracy, as displayed by the pairwise comparisons, indicates that this may have been due to the sample being underpowered. Although the improvements to accuracy that were observed were minor, this was likely due to the participants being healthy adults. Therefore, with the double-blinded, counterbalanced nature of the study reducing bias error, the indications of a positive effect from tRNS supports its practical applicability within a clinical context.

## Conclusion

The present study warrants that tRNS remains a promising tool for interventions aiming to improve numerical acuity, even after a single session. The bilateral IPS continues to be the most appropriate target region for stimulation to improve accuracy on tasks relying on non-verbal numerical processes. While primary statistical analyses did not reveal a significant interaction between tRNS and improvement in the MNL, pairwise comparisons revealed promising patterns for both the effect of a single session of tRNS on performance and in learning retention. Our findings have implications for educational and cognitive rehabilitation by informing which sites should be targeted during tRNS. Future studies are advised to consider the effects of baseline abilities and stimulation parameters on tRNS training programmes for those with developmental dyscalculia in order to ensure its effectiveness on numerical skills.

## Supplementary Information

Below is the link to the electronic supplementary material.

Supplementary Material 1

## Data Availability

The pre-registration as well as the datasets generated during and/or analysed during the current study are available in the OSF repository, The pre-registration as well as the datasets generated during and/or analysed during the current study are available in the OSF repository, [https://osf.io/7z9be/files/osfstorage?view_only=bd3e6aaf8bed4d25ba92fcc2e3b7d6bb%5D(https%3A%2Fosf.io%2F7z9be%2F%3Fview_only%3Dbd3e6aaf8bed4d25ba92fcc2e3b7d6bb] (https:/osf.io/7z9be/files/osfstorage?view_only=bd3e6aaf8bed4d25ba92fcc2e3b7d6bb).

## References

[1] Cisek, P. Resynthesizing behavior through phylogenetic refinement. *Atten. Percept. Psychophys.* **81**, 2265–2287. 10.3758/s13414-019-01760-1 (2019). 31161495 10.3758/s13414-019-01760-1 PMC6848052

[2] Agrillo, C. & Bisazza, A. Understanding the origin of number sense: A review of fish studies. *Philos. Trans. R. Soc. B: Biol. Sci.* **373**, 20160511. 10.1098/rstb.2016.0511 (2018). 10.1098/rstb.2016.0511 PMC5784038 29292358

[3] Rugani, R., Kelly, D. M., Szelest, I., Regolin, L. & Vallortigara, G. Is it only humans that count from left to right?. *Biol. Lett.* **6**, 290–292. 10.1098/rsbl.2009.0960 (2010). 20071393 10.1098/rsbl.2009.0960 PMC2880063

[4] Leibovich, T., Katzin, N., Harel, M. & Henik, A. From, “sense of number” to “sense of magnitude”: The role of continuous magnitudes in numerical cognition. *Behav. Brain Sci.* **40**, e164. 10.1017/S0140525X16000960 (2017). 27530053 10.1017/S0140525X16000960

[5] Lazzaro, G. et al. Understanding the effects of transcranial electrical stimulation in numerical cognition: A systematic review for clinical translation. *J. Clin. Med.* **11**, 2082. 10.3390/jcm11082082 (2022). 35456176 10.3390/jcm11082082 PMC9032363

[6] Fertonani, A., Pirulli, C. & Miniussi, C. Random noise stimulation improves neuroplasticity in perceptual learning. *J. Neurosci.* **31**, 15416–15423. 10.1523/JNEUROSCI.2002-11.2011 (2011). 22031888 10.1523/JNEUROSCI.2002-11.2011 PMC6703532

[7] Cappelletti, M. et al. Transfer of cognitive training across magnitude dimensions achieved with concurrent brain stimulation of the parietal lobe. *J. Neurosci.* **33**, 14899–14907. 10.1523/JNEUROSCI.1692-13.2013 (2013). 24027289 10.1523/JNEUROSCI.1692-13.2013 PMC3771029

[8] Bieck, S. M., Artemenko, C., Moeller, K. & Klein, E. Low to no effect: Application of tRNS during two-digit addition. *Front. Neurosci.* **12**, 176. 10.3389/fnins.2018.00176 (2018). 29674948 10.3389/fnins.2018.00176 PMC5895770

[9] Popescu, T. et al. Transcranial random noise stimulation mitigates increased difficulty in an arithmetic learning task. *Neuropsychologia* **81**, 255–264. 10.1016/j.neuropsychologia.2015.12.028 (2016). 26731199 10.1016/j.neuropsychologia.2015.12.028 PMC4749538

[10] Cappelletti, M., Pikkat, H., Upstill, E., Speekenbrink, M. & Walsh, V. Learning to integrate versus inhibiting information is modulated by age. *J. Neurosci.* **35**, 2213–2225. 10.1523/JNEUROSCI.1018-14.2015 (2015). 25653376 10.1523/JNEUROSCI.1018-14.2015 PMC6705357

[11] Hubbard, E. M., Piazza, M., Pinel, P. & Dehaene, S. Interactions between number and space in parietal cortex. *Nat. Rev. Neurosci.* **6**, 435–448. 10.1038/nrn1684 (2005). 15928716 10.1038/nrn1684

[12] Sokolowski, H. M., Fias, W., Mousa, A. & Ansari, D. Common and distinct brain regions in both parietal and frontal cortex support symbolic and nonsymbolic number processing in humans: A functional neuroimaging meta-analysis. *Neuroimage* **146**, 376–394. 10.1016/j.neuroimage.2016.10.028 (2017). 27769786 10.1016/j.neuroimage.2016.10.028

[13] Paulsen, D. J., Woldorff, M. G. & Brannon, E. M. Individual differences in nonverbal number discrimination correlate with event-related potentials and measures of probabilistic reasoning. *Neuropsychologia* **48**, 3687–3695. 10.1016/j.neuropsychologia.2010.08.014 (2010). 20817003 10.1016/j.neuropsychologia.2010.08.014 PMC2975800

[14] Pasqualotto, A. Transcranial random noise stimulation benefits arithmetic skills. *Neurobiol. Learn. Mem.* **133**, 7–12. 10.1016/j.nlm.2016.05.004 (2016). 27224886 10.1016/j.nlm.2016.05.004

[15] Snowball, A. et al. Long-term enhancement of brain function and cognition using cognitive training and brain stimulation. *Curr. Biol.* **23**, 987–992. 10.1016/j.cub.2013.04.045 (2013). 23684971 10.1016/j.cub.2013.04.045 PMC3675670

[16] Emerson, R. W. & Cantlon, J. F. Continuity and change in children’s longitudinal neural responses to numbers. *Dev. Sci.* **18**, 314–326. 10.1111/desc.12215 (2015). 25051893 10.1111/desc.12215 PMC4303560

[17] Faye, A. et al. Numerical cognition: A meta-analysis of neuroimaging, transcranial magnetic stimulation and brain-damaged patients studies. *NeuroImage: Clin.* **24**, 102053. 10.1016/j.nicl.2019.102053 (2019). 31795045 10.1016/j.nicl.2019.102053 PMC6978218

[18] Coolen, I., Judd, N., & Kievit, R. The Approximate number sense is (little more than) spatial skills. https://osf.io/preprints/psyarxiv/puvq5_v2 (2025).

[19] Kanjlia, S., Feigenson, L. & Bedny, M. Numerical cognition is resilient to dramatic changes in early sensory experience. *Cognition* **179**, 111–120. 10.1016/j.cognition.2018.06.004 (2018). 29935427 10.1016/j.cognition.2018.06.004 PMC6701182

[20] Lafay, A., St-Pierre, M. C. & Macoir, J. The mental number line in dyscalculia: Impaired number sense or access from symbolic numbers?. *J. Learn. Disabil.* **50**, 672–683. 10.1177/0022219416640783 (2017). 27015671 10.1177/0022219416640783

[21] Leibovich, T. & Ansari, D. The symbol-grounding problem in numerical cognition: A review of theory, evidence, and outstanding questions. *Can. J. Exp. Psychol/Revue Canadienne de Psychologie Expérimentale.* **70**, 12. 10.1037/cep0000070 (2016). 26913782 10.1037/cep0000070

[22] Von Aster, M. G. & Shalev, R. S. Number development and developmental dyscalculia. *Dev. Med. Child Nfeurol.* **49**, 868–873. 10.1111/j.1469-8749.2007.00868.x (2007). 10.1111/j.1469-8749.2007.00868.x 17979867

[23] Kucian, K. et al. Mental number line training in children with developmental dyscalculia. *Neuroimage* **57**, 782–795. 10.1016/j.neuroimage.2011.01.070 (2011). 21295145 10.1016/j.neuroimage.2011.01.070

[24] Izard, V. & Dehaene, S. Calibrating the mental number line. *Cognition* **106**, 1221–1247. 10.1016/j.cognition.2007.06.004 (2008). 17678639 10.1016/j.cognition.2007.06.004

[25] Leonard, S. J., Roche, C., Durkan, A., Gomides, M. & Santos, F. H. Children grow upwards, and so does the number line: Evidence from a directional number line paradigm. *Prog. Brain Res.* **279**, 37–56. 10.1016/bs.pbr.2023.03.002 (2023). 37661162 10.1016/bs.pbr.2023.03.002

[26] Schneider, M. et al. Associations of number line estimation with mathematical competence: A meta-analysis. *Child Dev.* **89**, 1467–1484. 10.1111/cdev.13068 (2018). 29637540 10.1111/cdev.13068

[27] Link, T., Moeller, K., Huber, S., Fischer, U. & Nuerk, H. C. Walk the number line—An embodied training of numerical concepts. *Trends Neurosci. Educ.*10.1016/j.tine.2013.06.005 (2013).

[28] Szczygieł, M. & Pieronkiewicz, B. Exploring the nature of math anxiety in young children: Intensity, prevalence, reasons. *Math. Think. Learn.* **24**, 248–266. 10.1080/10986065.2021.1882363 (2022).

[29] Ashcraft, M. H. & Krause, J. A. Working memory, math performance, and math anxiety. *Psychon. Bull. Rev.* **14**, 243–248. 10.3758/BF03194059 (2007). 17694908 10.3758/bf03194059

[30] Rossi, S. et al. Safety and recommendations for TMS use in healthy subjects and patient populations, with updates on training, ethical and regulatory issues: Expert guidelines. *J. Clinph.* **132**, 269–306 (2021). 10.1016/j.clinph.2020.10.003 PMC9094636 33243615

[31] Antal, A. et al. Low intensity transcranial electric stimulation: Safety, ethical, legal regulatory and application guidelines. *Clin. Neurophysiol. Off. J. Int. Fed. Clin. Neurophysiol.* **128**, 1774–1809. 10.1016/j.clinph.2017.06.001 (2017). 10.1016/j.clinph.2017.06.001 PMC5985830 28709880

[32] Leonard, S.J., Santos, F.H. Diverging roles of domain-specific anxieties in number-space associations. Insights from a multi-directional number line paradigm. *Psychological Research* **89**, 149 10.1007/s00426-025-02179-0 (2025). 10.1007/s00426-025-02179-0 PMC12484368 41026258

[33] Sasanguie, D., De Smedt, B. & Reynvoet, B. Evidence for distinct magnitude systems for symbolic and non-symbolic number. *Psycholog. Res.* **81**, 231–242. 10.1007/s00426-015-0734-1 (2017). 10.1007/s00426-015-0734-1 26708496

[34] Sasanguie, D., Göbel, S. M., Moll, K., Smets, K. & Reynvoet, B. Approximate 105 number sense, symbolic number processing, or number–space mappings: What underlies mathematics achievement?. *J. Exp. Child Psychol.* **114**, 418–431. 10.1016/j.jecp.2012.10.012 (2013). 23270796 10.1016/j.jecp.2012.10.012

[35] Gebuis, T. & Reynvoet, B. Generating nonsymbolic number stimuli. *Behav. Res. Methods* **43**(981–986), 2011. 10.3758/s13428-011-0097-5 (2011). 10.3758/s13428-011-0097-5 21512872

[36] DeWind, N. K., Adams, G. K., Platt, M. L. & Brannon, E. M. Modeling the approximate number system to quantify the contribution of visual stimulus features. *Cognition* **142**, 247–265. 10.1016/j.cognition.2015.05.016 (2015). 26056747 10.1016/j.cognition.2015.05.016 PMC4831213

[37] Hopko, D. R., Mahadevan, R., Bare, R. L. & Hunt, M. K. The abbreviated math anxiety scale (AMAS): Construction, validity, and reliability. *Assessment* **10**, 178–182. 10.1177/1073191103010002008 (2003). 12801189 10.1177/1073191103010002008

[38] Primi, C., Busdraghi, C., Tomasetto, C., Morsanyi, K. & Chiesi, F. Measuring math anxiety in Italian college and high school students: validity, reliability and gender invariance of the Abbreviated Math Anxiety Scale (AMAS). *J Lindif.* **34**, 51–56 (2014).

[39] Artemenko, C., Sitnikova, M. A., Soltanlou, M., Dresler, T. & Nuerk, H.-C. Functional lateralization of arithmetic processing in the intraparietal sulcus is associated with handedness. *Sci. Rep.*10.1038/s41598-020-58477-7 (2020). 32020021 10.1038/s41598-020-58477-7 PMC7000739

[40] Artemenko, C. et al. Differences in math ability determine neurocognitive processing of arithmetic complexity: A combined fNIRS-EEG study. *Front. Hum. Neurosci.*10.3389/fnhum.2019.00227 (2019). 31333436 10.3389/fnhum.2019.00227 PMC6616314

[41] Stoet, G. PsyToolkit: A software package for programming psychological experiments using Linux. *Behav. Res. Methods* **42**, 1096–1104 (2010). 21139177 10.3758/BRM.42.4.1096

[42] Stoet, G. PsyToolkit: A novel web-based method for running online questionnaires and reaction-time experiments. *Teach. Psychol.* **44**, 24–31 (2017).

[43] Karolis, V. R. et al. Probing the architecture of visual number sense with parietal tRNS. *Cortex* **114**, 54–66. 10.1016/j.cortex.2018.08.030 (2019). 30316449 10.1016/j.cortex.2018.08.030

[44] Hussain, M., Davis, N. J. & Benn, Y. A single tDCS session can enhance numerical competence. *Neuropsychologia* **193**, 108760. 10.1016/j.neuropsychologia.2023.108760 (2024). 38103681 10.1016/j.neuropsychologia.2023.108760

[45] Hartwright, C. E. et al. The neurocognitive architecture of individual differences in math anxiety in typical children. *Sci. Rep.* **8**, 8500. 10.1038/s41598-018-26912-5 (2018). 29855608 10.1038/s41598-018-26912-5 PMC5981328

[46] Sarkar, A., Dowker, A. & Cohen Kadosh, R. Cognitive enhancement or cognitive cost: Trait-specific outcomes of brain stimulation in the case of mathematics anxiety. *J. Neurosci.* **34**, 16605–16610. 10.1523/JNEUROSCI.3129-14.2014 (2014). 25505313 10.1523/JNEUROSCI.3129-14.2014 PMC4261089

[47] Arsalidou, M. & Taylor, M. J. Is 2 + 2 = 4? Meta-analyses of brain areas needed for numbers and calculations. *Neuroimage* **54**, 2382–2393. 10.1016/j.neuroimage.2010.10.009 (2011). 20946958 10.1016/j.neuroimage.2010.10.009

[48] Kucian, K. et al. Impaired neural networks for approximate calculation in dyscalculic children: A functional MRI study. *Behav. Brain Funct.: BBF.* **2**, 31. 10.1186/1744-9081-2-31 (2006). 16953876 10.1186/1744-9081-2-31 PMC1574332

[49] Iuculano, T. & Cohen Kadosh, R. Preliminary evidence for performance enhancement following parietal lobe stimulation in developmental Dyscalculia. *Front. Hum. Neurosci.*10.3389/fnhum.2014.00038 (2014). 24570659 10.3389/fnhum.2014.00038 PMC3916771

[50] Davis, N. J. Variance in cortical depth across the brain surface: Implications for transcranial stimulation of the brain. *Eur. J. Neurosci.* **53**, 996–1007. 10.1111/ejn.14957 (2021). 32877015 10.1111/ejn.14957

[51] Looi, C. Y. et al. Combining brain stimulation and video game to promote long-term transfer of learning and cognitive enhancement. *Sci. Rep.* **6**, 22003. 10.1038/srep22003 (2016). 26902664 10.1038/srep22003 PMC4763231

[52] Iuculano, T. & Cohen Kadosh, R. The mental cost of cognitive enhancement. *J. Neurosci.* **33**, 4482–4486. 10.1523/JNEUROSCI.4927-12.2013 (2013). 23467363 10.1523/JNEUROSCI.4927-12.2013 PMC3672974

[53] Looi, C. Y. et al. Transcranial random noise stimulation and cognitive training to improve learning and cognition of the atypically developing brain: A pilot study. *Sci. Rep.* **7**, 4633. 10.1038/s41598-017-04649-x (2017). 28680099 10.1038/s41598-017-04649-x PMC5498607

