# Supplementary material for: Effects on nonverbal numerical acuity performance after a single-session transient random noise stimulation over the intraparietal sulcus or dorsolateral prefrontal cortex
Source: Sci Rep. 2025 Oct 30;15:37977. doi: 10.1038/s41598-025-21890-x (PMC12575817; doi:10.1038/s41598-025-21890-x)
Supplement: Supplementary file 1 — Supplementary Material 1 [file 41598_2025_21890_MOESM1_ESM.pdf]

Table 1

Table of Skewness and kurtosis for treatment condition subgroups for number line estimation task

Treatment condition	Measure	Treatment	Skewness	Kurtosis
A	Accuracy	Sham_Pre	-.255	-.506
		Sham_Post	.058	-1.051
		tRNS_Pre	.032	-.174
		tRNS_Post	-.626	2.673
	Reaction time	Sham_Pre	.325	.486
		Sham_Post	.884	.654
		tRNS_Pre	1.263	1.995
		tRNS_Post	1.176	2.300
B	Accuracy	Sham_Pre	-1.378	2.970
		Sham_Post	-.243	-1.181
		tRNS_Pre	-.738	-.511
		tRNS_Post	-.045	-1.083
	Reaction time	Sham_Pre	.215	-1.1
		Sham_Post	1.043	.572
		tRNS_Pre	.388	-.399
		tRNS_Post	-.209	-.648

Table 2

Table of Skewness and Kurtosis for brain area subgroups for number line estimation task.

Brain area	Measure	Treatment	Skewness	Kurtosis
Parietal	Accuracy	Sham_Pre	-.978	1.114
		Sham_Post	.364	-1.124
		tRNS_Pre	-.282	-.710
		tRNS_Post	-.215	.436
	Reaction time	Sham_Pre	1.007	1.275
		Sham_Post	2.253	6.108
		tRNS_Pre	-.398	-.266
		tRNS_Post	.-.258	.041
Frontal	Accuracy	Sham_Pre	.015	-.896
		Sham_Post	-.471	-.649
		tRNS_Pre	-.396	.363
		tRNS_Post	-.335	-.703
	Reaction time	Sham_Pre	-.072	2.369
		Sham_Post	.419	.874
		tRNS_Pre	.443	-.981
		tRNS_Post	.544	.1345
